# Sex differences in laryngeal cancer treated with CO_2-_transoral laser microsurgery: a case-control study

**DOI:** 10.1007/s00405-025-09315-x

**Published:** 2025-03-14

**Authors:** Isabel Vilaseca, C. Sampieri, E. Lehrer, R. D. Ramírez, J. M. Costa, I. Valduvieco, N. Basté, S. Medrano, Y. Rodriguez, A. Muxí, F. X. Avilés-Jurado, M. Bernal-Sprekelsen

**Affiliations:** 1 Otorhinolaryngology Department, Hospital Clínic, Barcelona, Spain; 2 Surgical Area. Hospital Clínic, Barcelona, Spain; 3 Head Neck Cancer Unit, Hospital Clínic, Barcelona, Spain; 4 Department of Surgery and Medical-Surgical Specialties, Faculty of Medicine, Universitat de Barcelona, Barcelona, Spain; 5 Translational genomics and targeted therapies in solid tumours. Institut d´Investigacions Biomèdiques August Pi i Sunyer, IDIBAPS, Barcelona, Spain; 6 Agència de Gestió d’Ajuts Universitaris i de Recerca, Barcelona, Spain; 7 Department of Experimental Medicine (DIMES), University of Genova, Genova, Italy; 8 Imatge Molecular en Medicina Nuclear, Agència de Gestió d’Ajuts Universitaris i de Recerca, Barcelona, Spain; 9 Otorhinolaryngology Department & Surgical Area, Hospital Clínic, Villarroel,170, Barcelona, 08036 Spain

**Keywords:** Larynx cancer, Transoral laser microsurgery, Propensity score, Survival outcomes, Gender disparity

## Abstract

**Background:**

The aim of the present study was to evaluate the sex-disparities in larynx cancer patients treated with CO_2-_Transoral Laser Microsurgery (CO_2_-TOLMS).

**Methods:**

Retrospective analysis of 1290 consecutive patients. Oncologic and functional outcomes were evaluated according to sex groups. Survival rates were compared by propensity-score matching approach and multivariable Cox regression analysis.

**Results:**

Among 1290 patients, 122 (9.5%) were females. No significant differences were observed in tumor exposure, margins or pathology characteristics. Women experienced fewer complications than men (0.12 vs. 0.06; *p* = 0.007). There were neither significant differences in voice or swallowing outcomes, nor in the need for tracheostomy or gastrostomy. 5-y disease-free (63% vs. 66%, *p* = 0.92), 5-y disease-specific (91% vs. 93%, *p* = 0.54) and 5-y overall survival (78% vs. 84%, *p* = 0.18) rates were not different between men and woman.

**Conclusions:**

CO_2_-TOLMS is a valid treatment for larynx cancer in women, with equivalent outcomes than those achieved in the male population.

## Introduction

Classically, an increased risk and incidence of some types of cancer has been described in men, with a lower survival rate than women [1]. Among these, are head and neck squamous cell cancers (HNSCC), which have traditionally been linked to tobacco and alcohol intake.

Laryngeal cancer (LC) is one of the most prevalent entities of head and neck cancer, with an estimated incidence of around 5.8 cases per 100,000 in males compared to 1.2 per 100,000 in women [2]. Wang et al. analyzed the temporal trends in incidence and mortality rates of laryngeal cancer during the period 1990–2017 at the global, regional and national levels, and observed a high geographical heterogeneity [3]. There were significant decreasing trends in the incidence and mortality rates globally, mainly due to the efficacy of prevention strategies, but they also observed unfavourable trends in a few countries located in East Asia and North Africa. Owing to the large population sizes in these countries, globally, the incidence and deaths due to LC increased 58.7% and 33.9%, respectively [3]. Most deaths occurred in men and in regions with low-to-medium social demographic indexes. Overall, the patterns of incidence were similar between the sexes, although men consistently presented with higher numbers and rates than women [4].

Several risk factors have been related to the pathogenesis of laryngeal cancer, being the most relevant tobacco use and alcohol consumption. In Western European countries, due to the progressive promotion of gender equality and female empowerment, the number of female smokers had increased significantly by the end of last century, before measures aimed at reducing tobacco use were established. As a result, the incidence of laryngeal and hypopharyngeal cancers has currently increased in women in some countries, narrowing slightly the prevalence gap between genders for this neoplasm [5].

The relationship between tobacco use and laryngeal cancer is linear, but is enhanced by alcohol consumption. In fact, Europe, where alcohol consumption has been higher than the global average, still has a high incidence of LC [6]. From this point of view and although some measures have been implemented to reduce alcohol use in recent years, we are seeing a new increase in consumption, an increase that is estimated to be maintained until 2030 [7]. This finding may represent an alarm for the maintenance of laryngeal cancer and other diseases associated with alcohol in the near future.

Apart from tobacco and alcohol consumption, recent studies suggest that males are much more susceptible to head and neck cancers than females, being these differences more evident in the larynx and hypopharynx. Park et al. [8] evaluated 9,598,085 individuals who underwent regular health checkups in Korea and sought for head and neck cancers developed during a period of 10 years. The incidence rate was 0.19 in males (8.500 affected) and 0.06 in females (2.232 affected). Notably, the male–female ratio increased with age below 70 years, but decreased thereafter. When never-smokers and never-drinkers (only) were compared, males remained at a 2.9-fold higher risk of head and neck cancer than females. The authors suggested that hormone control of cancer progression could be a mechanism, as the larynx is a secondary sex organ that undergoes physiological changes during puberty, and laryngeal cancer response to 17β-estradiol via the estrogen receptor has been correlated with a better survival in the literature [9, 10]. Moreover, higher expressions of estrogen and progesterone receptors have been related with aggressive clinical and pathological manifestations [11].

Regarding the treatment outcomes, little is known about the peculiarities and differences between men and women submitted to larynx cancer treatment, because experience in the past has been limited by its low incidence in females [3]. A recent meta-analysis that included a total of 9.161 patients with laryngeal cancer (86.9% males and 13.0% females) tried to investigate the prognosis of sex in laryngeal cancer survival [12]. The authors concluded that sex did not appear to be an independent factor affecting overall, disease-free or disease-specific survivals, although they advised of the lack of sufficient survival data in studies published over the past century.

In current international guidelines, curative treatment options for larynx cancer are identical for male and female patients, and no specific considerations are mentioned when different treatment alternatives are available (13–14). Exploring sex disparities in all the treatment modalities would be of interest. One of the more spread larynx cancer treatments is CO_2_-transoral laser microsurgery (CO_2_-TOLMS), a standardized minimally invasive treatment recommended for early and intermediate laryngeal cancer [15–17]. In CO_2_-TOLMS, adequate exposure to the larynx is necessary to achieve a complete resection of the tumor, which is essential to obtain adequate oncologic and functional outcomes. On the other hand, it is well known that the male larynx is morphologically different from the female, larger and with a more prominent Adam’s apple. As the larynx enlarges in puberty, this anterior segment further enlarges in males, lengthening the true vocal folds and reducing the anterior angle of the thyroid cartilage [18]. Thus, one can hypothesize that direct laryngoscopy during transoral resection may be more difficult in women (smaller larynx) and even have an impact on tumor exposure, treatment accomplishment, and need of additional treatment, not to mention potential side effects such as stenosis or deglutition problems. Few studies have specifically evaluated the outcomes of this minimally transoral approach in the female population.

The purpose of the present study was to evaluate the differences in treatment outcomes between female and male larynx cancer patients treated with CO_2_-TOLMS.

## Materials and methods

Retrospective analysis of prospectively collected data. All consecutive patients treated of laryngeal cancer with CO_2_-TOLMS from 1998 to 2022 at a tertiary academic center were included. In 1998, CO_2_-TOLMS was established as the treatment of choice for early and intermediate laryngeal cancer at our center, shifting from open to transoral approaches. Since then, with minor modifications, it has remained the first-line treatment for most patients. The present study was conducted in accordance with the Declaration of Helsinki and was approved by the local ethics committee with number HCB/2015/0733. Due to its retrospective nature, the patients did not specifically sign any informed consent.

Patients were evaluated in a multidisciplinary tumor board and staged according to the 2017 TNM classification proposed by AJCC-UICC [19]. For the present analysis patients previously treated with surgery or radiotherapy or patients previously treated for any other head and neck cancer were excluded, as were patients with distant metastasis at presentation.

In our protocol, CO_2_-TOLMS is recommended as a first-line option independendently of age and sex in early and selected intermediate laryngeal cancers. Moreover, the information provided to patients in the tumor board is exactly the same, regardless of gender. T4 cases with thyroid cartilage infiltration or limited extralaryngeal extension were included in the early days of TOLMS, but excluded latter on due to lower outcomes compared to other treatment alternatives. Tumors with extralaryngeal growth, extensive infiltration of the thyroid cartilage, cricoid involvement, deep or bilateral invasion of the posterior commissure, or extensive invasion of the subglottis are excluded from the indication for CO_2_-TOLMS. Some of the selected T4a tumors included in the present series, were staged before 2002 and would not be selected for TOLMS nowadays.

The detailed description of the surgical technique has been previously published elswhere [20]. In summary, small tumors were removed in monoblock whereas in intermediate tumors a blockwise resection was performed. Exposure was considered adequate when a good view of the tumor was achieved with the large bore laryngoscope, either with or without external compression or flexion-extension of the head. In contrast, when this was not the case and a small bore laryngoscope was required for the major part of the surgery, exposure was considered difficult.

Transoral surgery was followed by a neck dissection in clinically or radiologically positive neck nodes. In general, all glottic tumors and T1-T2 supraglottic tumors with radiologically negative necks did not undergo neck dissection and were closely followed up with ultrasonography or CT for the first two years.

Resection margins were classified as free of tumor, close/uncertain (non-assessable or with tumor nests at less than 2 mm) or positive according to Blanch et al. [21]. In the case of positive margins, additional surgery was proposed to enlarge the resection. When this was not possible (tumor reached the cartilage) or the patient refused, additional treatment with open surgery or radiotherapy was proposed. Adjuvant treatment administration was discussed at the tumor board in case of > 1 positive lymph node, presence of extranodal spread or in tumors with vascular, lymphatic or perineural infiltration.

For functional assessment, the need for nasogastric tube after surgery (days), gastrostomy (never-temporary-permanent) and tracheostomy (never-temporary-permanent) were systematically registered in our database.

The degree of postoperative dysphonia was assessed using a scale ranging from 1 to 3 as follows: (1) normal or near-normal voice (abnormalities would only be detected by specially trained personnel), (2) mild dysphonia (useful voice; the patient is frequently asked about the voice problem and has to give explanations, or the patient has intermittent dysphonia), (3) severe dysphonia (voice like persistent laryngitis, not useful).

Swallowing was assessed by anamnesis and flexible endoscopic evaluation 6 months after finishing treatment. Variables included the presence of cough during oral intake (no/yes), repeated aspiration pneumonia (no/yes), need for gastrostomy or tracheostomy secondary to aspirations (never/temporary/permanent), or total laryngectomy for functional reasons.

### Statistical analysis

Data are presented as a mean (SD), median (IQR) or as a number (percentage). Variables studied included age, sex (male/female), tobacco and alcohol habit (no/yes), diabetes (no/yes), year of treatment, location (supraglottic/glottic), pT category, pN category, margins (free/affected/uncertain), postoperative adjuvant treatment (no/yes), number of complications, length of hospitalization (days), days of feeding tube, voice outcome, aspiration signs, need of tracheotomy and need of gastrostomy.

Variables were compared according to sex groups and sublocation of the tumor in the larynx. Comparisons were assessed with Pearson´s chi squared method, T-test or non-parametric tests according to distribution.

Overall survival (OS), disease-specific survival (DSS) and disease-free survival (DFS) were estimated using the Kaplan–Meier method. OS was assessed from the date of surgery to the date of death (regardless of the cause) or the date of the last consultation for censored observations. To calculate DSS, only deaths related to the tumor or complications of the treatment were considered. Events for DFS were defined as the presence of cancer recurrence or death due to any cause. Comparisons in survival rates between groups were assessed with the Log-rank test.

A propensity-score matching was applied to obtain an unbiased estimate of survivals to compare males vs. females [22]. In the available dataset, 0.6% of the values were missing and were imputed with a MICE algorithm [23]. No follow-up data was missing. Based on literature-derived knowledge, on differences found between males and females in the univariate analysis, and on clinical expertise, the following variables were taken into account for matching: age, smoking habit, alcohol abuse, pT, pN, period of treatment, and tumor localization. These variables were chosen to estimate the propensity-score. This score was calculated using a logistic regression model based on the observed baseline covariates. The optimal pair-matching method was applied to match male patients to similar female patients with 1:1 ratio (24–25). The calipers were set with a width equal to 0.2 of the standard deviation of the logit of the propensity-score. Visual plots and standardized mean differences were used to assess the balance of covariates and were compared before and after the matching to evaluate if potential confounders were well-balanced between the two study groups. The two-sample Wilcoxon rank-sum test was used to test the hypothesis of equal distributions among the two groups for continuous variables. For categorical variables, the proportions in the two groups were compared using the Chi-square or Fisher’s exact test, as appropriate.

Independent factors for OS, DFS, DSS and total laryngectomy free survival (TLFS) were additionally explored for the whole group in a multivariable Cox regression model through backwards selection.

Data were analyzed using RStudio. A p value of 0.05 was defined as statistically significant. Data reporting followed the STROBE (STrengthening the Reporting of OBservational studies in Epidemiology) guidelines (Supplementary File S1).

## Results

**Fig. 1:**
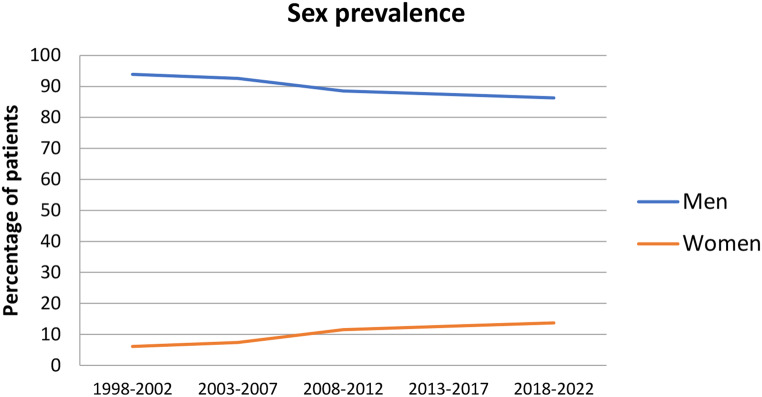
Evolution of sex prevalence in the cohort of patients treated with CO_2_-TOLMS

The complete sample included 1.290 consecutive patients of which 122 (9.5%) were female. The incidence of larynx cancer treated with CO_2_-TOLMS in the women group increased slightly over time, being the opposite in the male group. The prevalence also showed a progressive increase in women, which doubled the initial rate in the last years (Fig. 1).

The mean age of the patients at the time of CO_2_-TOLMS was 64.4 (11.1) years. Women were younger than men (62.6 vs. 64.6; *p* = 0.036), had been less exposed to tobacco (never smoker 37% vs. 5%; *p* < 0.001) and alcohol consumption (never drinker 77.1% vs. 51% *p* < 0.001), and presented a higher rate of supraglottic tumors (36.8% vs. 25%; *p* = 0.004). Overall, there were no significant differences in vocal cord motility, tumor exposure, histopathology or definitive margins for each of the sublocations in the larynx. However, men were more likely to present larger local extension in the glottis (15,9% vs. 7.7%; *p* = 0.05) and a higher rate of nodal involvement in the supraglottis (37.6% vs. 15.9%; *p* = 0.005). The characteristics of the sample are expressed in Table 1.

**Table 1:** Characteristics of the patients according to sex group and sublocation in the larynx

	Supraglottis			Glottis		
	Men	Women	*p-*value	Men	Women	*p-*value
	N (%)	N (%)		N (%)	N (%)	
Age (years) *			**0.025**			0.571
	62.7 (9.9)	59 (12.4)		65.3 (11)	64.3 (14.3)	
Age group			0.497			0.991
≤ 70	236 (79.7)	37 (84.1)		565 (64.9)	50 (54.9)	
> 70	60 (20.3)	7 (15.9)		306 (35.1)	27 (35.1)	
Tobacco consumption			**< 0.001**			**< 0.001**
Never	4 (1.4)	6 (13.6)		50 (5.8)	38 (50)	
Yes	287 (98.6)	38 (86.4)		814 (94.2)	38 (50)	
Tobacco Index (p/year) *			**< 0.001**			**< 0.001**
	57.2 (28.8)	36.3 (25.9)		45.5 (27.6)	15.4 (22.3)	
Alcohol intake			**0.002**			**< 0.001**
Never	111 (38.5)	28 (63.6)		471 (55.2)	63 (85.1)	
Yes	177 (61.5)	16 (36.4)		383 (44.8)	11 (14.9)	
Diabetes			**0.028**			**0.005**
No	230 (79.3)	41 (93.2)		735 (86)	73 (97.3)	
Yes	60 (20.7)	3 (6.8)		120 (14)	2 (2.7)	
Vocal cord motility			0.537			0.198
Normal	252 (85.1)	39 (88.6)		721 (82.8)	69 (88.5)	
Altered	44 (14.9)	5 (11.4)		150 (17.2)	9 (11.5)	
Tumor exposure			0.36			0.465
Good	263(88.9)	37 (84.1)		745 (85.4)	69 (88.5)	
Difficult	33 (11.1)	7 (15.9)		127 (14.6)	9 (11.5)	
Pathology report			0.553			0.484
SCC	282 (95.3)	41 (93.2)		850 (97.5)	75 (96.2)	
Other	14 (4.7)	3 (6.8)		22 (2.5)	3 (3.8)	
Grade differentiation			0.955			0.809
I	11 (4.5)	1 (3.3)		85 (14.9)	4 (11.4)	
II	131 (53.5)	16 (53.3)		372 (65.3)	23 (65.7)	
III	103 (42)	13 (43.3)		113 (19.8)	8 (22.9)	
Margins status			0.363			0.957
Free of tumor	166 (56.1)	22 (50)		459 (52.9)	42 (54.5)	
Close/positive	130 (43.9)	22 (50)		409 (47.1)	35 (45.5)	
pT			0.327			**0.057**
Tis-T1-T1a-T1b	56 (18.9)	10 (22.7)		495 (56.8)	56 (71.8)	
T2	93 (31.4)	8 (18.2)		238 (27.3)	16 (20.5)	
T3	125 (42.2)	23 (52.3)		127 (14.6)	6 (7.7)	
T4	22 (7,4)	3 (6.8)		12 (1.4)	-	
Local extension			0.243			**0.052**
Early (T1-T2)	149 (50.3)	18 (40.9)		733 (84.1)	72 (92.3)	
Advanced (T3-T4)	147 (49.7)	26 (59.1)		139 (15.9)	6 (7.7)	
Nodal involvement			**0.005**			0.945
pN-/pNx	184 (62.4)	37 (84.1)		860 (98.6)	77 (98.7)	
pN+	111 (37.6)	8 (15.9)		12 (1.4)	1 (1.3)	
Stage			0.274			**0.002**
0	-	-		42 (4.8)	11 (14.1)	
I	49 (16.6)	9 (20.5)		453 (51.9)	45 (57.7)	
II	66 (22.3)	7 (15.9)		231 (26.5)	16 (20.5)	
III	94 (31.8)	20 (45.5)		133 (15.3)	6 (7.7)	
IV	84 (29.4)	8 (18.2)		13 (1.5)	-	
Adjuvant radiotherapy			**0.043**			0.187
No	205 (69.3)	37 (84.1)		857 (98.3)	75 (96.2)	
Yes	91 (30.7)	7 (15.9)		15 (1.7)	3 (3.8)	
Persistence of smoking			0.087			0.37
No	229 (77.4)	38 (86.4)		733 (84.1)	69 (88.5)	
Yes	37 (12.5)	6 (13.6)		100 (11.5)	8 (10.3)	
Unknown	30 (10.1)	-		39 (4.5)	1 (1.3)	

SCC: Squamous cell carcinoma; pN-: all the nodes were negative after neck dissection; pNx: neck dissection was not performed; pN+: there were histologically positive nodes in the neck dissection. * Data are expressed as mean and (standard deviation)

No significant differences were observed in terms of laryngeal exposure during surgery, margins status, or pathology report. By contrast, during the follow-up men presented a higher rate of local relapse in the case of glottic tumors that ended up in a higher total laryngectomy rate (14.4% vs., 5.1%; *p* = 0.22). (Table 2)

**Table 2:** Outcomes after CO2-TOLMS according to sex group and tumor location

	Supraglottis			Glottis		
	Men	Women	*p-*value	Men	Women	*p-*value
	N = 296	N = 44		N = 872	N = 78	
Feeding tube (days) *			0.793			0.608
	10.8 (9.6)	9.6 (± 12.1)		0.3(2.1)	0.5 (2.5)	
No. Complications *			**0.004**			**0.047**
	0.39 (0.5)	0.1 (± 0.3)		0.07 (0.3)	0.03 (0.2)	
Complication rate			0.129			0.397
No	222 (75)	39 (88.6)		817 (93.7)	76 (97.4)	
Yes	74 (25)	5 (11.4)		55 (6.3)	2 (2.6)	
Length hospital stay (days) *			**0.002**			**0.007**
	11 (9.8)	7.5 (6)		2.8 (2.1)	2.1 (2.1)	
Vocal outcome			0.378			0.306
Normal voice	205 (71.4)	27 (61.4)		279 (34.1)	32 (42.1)	
Mild dysphonia	56 (19.5)	11 (25)		334 (40.9)	25 (32.9)	
Severe dysphonia	26 (9.1)	6 (13.6)		204 (25)	19 (25)	
Aspiration			0.447			0.25
Never	243 (82.1)	40 (90.9)		842 (96.6)	78 (100)	
Occasional cough	43 (14.5)	4 (9.1)		28 (3.2)	0	
Repetitive Pneumonia	6 (2.0)	0		2 (0.2)	0	
Gastrostomy			0.762			1
Never	281 (94.9)	42 (95.5)		872 (100)	78 (100)	
Temporary	9 (3)	2 (4.5)		0		
Definitive	5 (1.7)	0		0		
Tracheotomy			0.392			0.392
Never	260 (88.4)	39 (88.6)		850 (97.5)	76 (97.4)	
Temporary	29 (9.9)	3 (6.8)		17 (1.9)	1 (1.3)	
Definitive	5 (1.7)	2 (4.5)		5 (0.6)	1 (1.3)	
Follow-up (months) *			0.215			0.933
	58.5 (43)	67 (37.3)		64.8 (42.4)	65.2 (± 45.4)	
Local relapse			0.645			**0.013**
No	224 (75.9)	32 (72.7)		637 (73.1)	67 (85.9)	
Yes	71 (24.1)	12 (27.3)		235 (26.9)	11 (14.1)	
Nodal relapse			0.607			0.427
No	253 (85.8)	39 (88.6)		833 (95.5)	76 (97.4)	
Yes	42 (14.2)	5 (11.4)		39 (4.5)	2 (2.6)	
Distant metastasis			0.236			0.2
No	266 (89.9)	42 (95.5)		854 (97.9)	78 (100)	
Yes	30 (10.1)	2 (4.5)		18 (2.1)	0	
Total laryngectomy			0.875			**0.022**
No	253 (85.5)	38 (86.4)		746 (85.6)	74 (94.9)	
Yes	43 (14.5)	6 (13.6)		126 (14.4)	4 (5.1)	

* Data are expressed as mean and (standard deviation)

Women experienced a reduced number of complications compared to men, in both, supraglottis (0.1% vs. 0.39%; *p* = 0.004) and glottis (0.03% vs. 0.07%; *p* = 0.047), with decreased hospital stay. From the functional point of view, there were neither significant differences in voice or swallowing outcomes after treatment, nor in the need for tracheostomy or gastrostomy during the post-operative period. (Table 2)

**Fig. 2:**
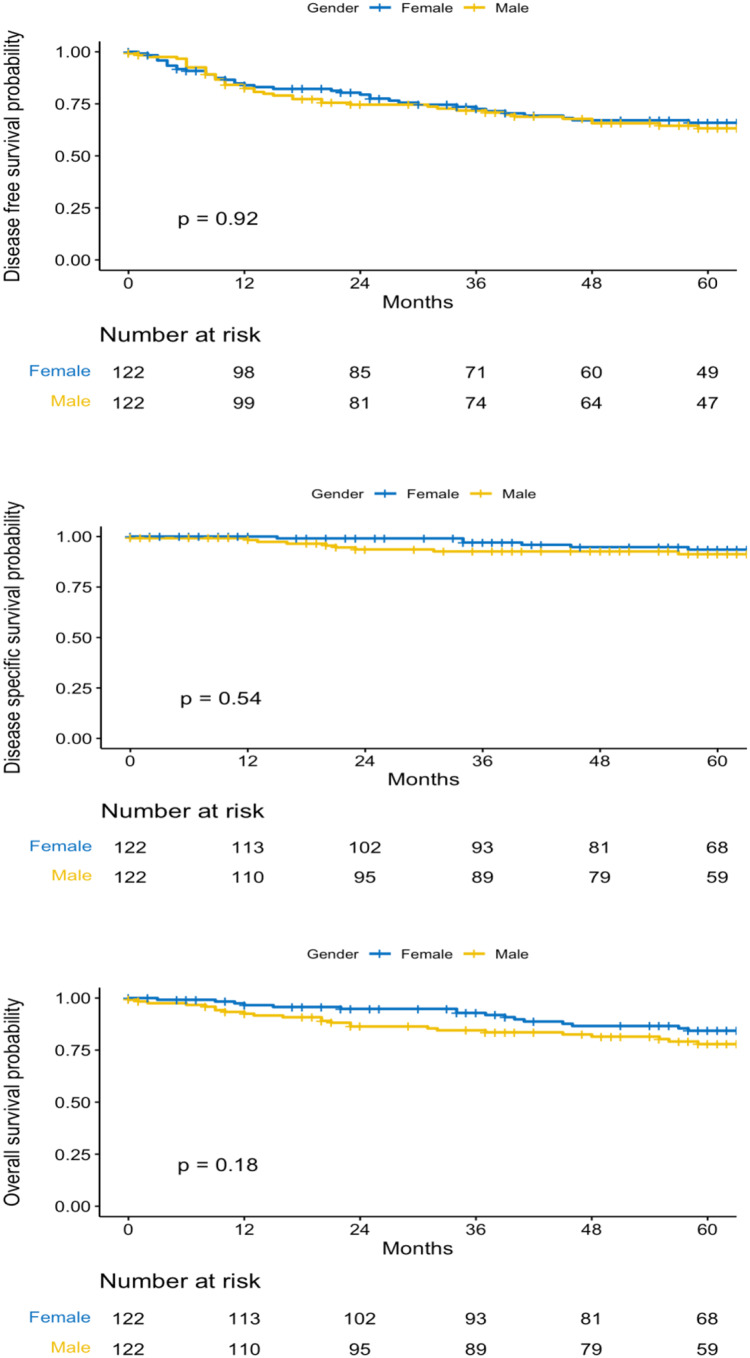
Survival of the patients according to sex group

The median follow-up of the patients was 60.7 months; 60.55 for male group and 62.32 for the female. The characteristics of the matched cohort of patients are expressed in Table 3. Five years disease-free survival (63% vs. 66%, *p* = 0.92), disease-specific survival (91% vs. 93%, *p* = 0.54) and overall survival (78% vs. 84%, *p* = 0.18) rates, were not significantly different between men and women (Fig. 2). At multivariable analysis, older age, initial period of intervention, alcohol abuse and T and N categories were independent factors for reduced OS, whereas positive margins status was for reduced DFS, DSS and TLFS. Glottic location independently increased the risk of total laryngectomy compared to supraglottic location. (Table 4)

**Table 3:** Characteristics of the matched cohort

Variable	Overall *N* = 244	Sex group		*p*-value
		Female *N* = 122	Male *N* = 122	
**Age**				0.34
Mean (SD)	61.77 (12.62)	62.46 (13.78)	61.07 (11.35)	
**Smoke**				0.89
Never	87 (36%)	44 (36%)	43 (35%)	
Yes	157 (64%)	78 (64%)	79 (65%)	
**Alcohol**				0.43
Never	193 (79%)	94 (77%)	99 (81%)	
Yes	51 (21%)	28 (23%)	23 (19%)	
**Diabetes**				**< 0.001**
No	217 (89%)	117 (96%)	100 (82%)	
Yes	27 (11%)	5 (4.1%)	22 (18%)	
**Localization**				0.29
Glottis	148 (61%)	78 (64%)	70 (57%)	
Supraglottis	96 (39%)	44 (36%)	52 (43%)	
**Exposition**				0.37
Difficult	37 (15%)	16 (13%)	21 (17%)	
Good	207 (85%)	106 (87%)	101 (83%)	
**Period treatment**				**0.011**
1998–2002	65 (27%)	21 (17%)	44 (36%)	
2003–2007	38 (16%)	22 (18%)	16 (13%)	
2008–2012	75 (31%)	38 (31%)	37 (30%)	
2013–2017	38 (16%)	24 (20%)	14 (11%)	
2018–2022	28 (11%)	17 (14%)	11 (9.0%)	
**T category**				0.85
T1	130 (53%)	66 (54%)	64 (52%)	
T2	53 (22%)	24 (20%)	29 (24%)	
T3	56 (23%)	29 (24%)	27 (22%)	
T4	5 (2.0%)	3 (2.5%)	2 (1.6%)	
**N category**				0.86
N0	226 (93%)	113 (93%)	113 (93%)	
N1	8 (3.3%)	3 (2.5%)	5 (4.1%)	
N2	9 (3.7%)	5 (4.1%)	4 (3.3%)	
N3	1 (0.4%)	1 (0.8%)	0 (0%)	
**Stage**				0.89
0	20 (8.2%)	11 (9.0%)	9 (7.4%)	
I	109 (45%)	54 (44%)	55 (45%)	
II	49 (20%)	23 (19%)	26 (21%)	
III	53 (22%)	26 (21%)	27 (22%)	
IV	13 (5.3%)	8 (6.6%)	5 (4.1%)	
**Radiotherapy**				> 0.99
No	224 (92%)	112 (92%)	112 (92%)	
yes	20 (8.2%)	10 (8.2%)	10 (8.2%)	
**Margins**				0.45
Free	137 (56%)	64 (52%)	73 (60%)	
Positive	15 (6.1%)	9 (7.4%)	6 (4.9%)	
Uncertain	92 (38%)	49 (40%)	43 (35%)	
**Number complications**				0.078
0	221 (91%)	115 (94%)	106 (87%)	
1	22 (9.0%)	7 (5.7%)	15 (12%)	
2	1 (0.4%)	0 (0%)	1 (0.8%)	

**Table 4:** Independent variables in the Cox regression multivariable analysis

	Hazard Ratio	95,0% CI		Sig.
		Lower	Upper	
**Total Laryngectomy free survival**				
**Age**	1.028	1.014	1.042	<.001
**Localization**				0.015
Supraglottis (reference)				
Glottis	1.55	1.089	2.207	0.015
**T category**				<.001
T1 (reference)				
T2	6.209	3.901	9.884	<.001
T3	11.138	6.9	17.979	<,001
T4	13.47	6.546	27.715	<,001
**Margins**				0.02
Negative (reference)				
Close/Uncertain	1.193	0.87	1.636	0.274
Positive	1.961	1.222	3.145	0.005
**Disease free survival**				
**Age**	1.036	1.027	1.044	<.001
**Alcohol**				0.011
Never (reference)				
Yes	1.255	1.054	1.494	0.011
**T category**				<.001
T1 (reference)				
T2	1.904	1.543	2.35	<.001
T3	2.424	1.928	3.047	<.001
T4	2.102	1.325	3.333	0.002
**N category**				0.002
N0/Nx (reference)				
N1	1.398	0.953	2.05	0.087
N2	1.727	1.252	2.382	<.001
N3	2.598	0.819	8.239	0.105
**Margins**				0.002
Negative (reference)				
Close/Uncertain	1.243	1.035	1.493	0.02
Positive	1.579	1.186	2.103	0.002
**Disease specific survival**				
**Age**	1.033	1.015	1.051	<.001
**Period of treatment**				0.017
1998–2002 (reference)				
2003–2007	0.743	0.474	1.166	0.197
2008–2012	0.466	0.268	0.81	0.007
2013–2017	0.417	0.215	0.807	0.009
2018–2022	0.32	0.098	1.049	0.06
**T category**				<.001
T1 (reference)				
T2	7.512	3.984	14.165	<.001
T3	10.793	5.672	20.539	<.001
T4	11.771	4.843	28.61	<.001
**N category**				<.001
N0/Nx (reference)				
N1	1.209	0.599	2.44	0.596
N2	2.833	1.774	4.523	<.001
N3	10.677	2.348	48.542	0.002
**Margins**				,098
Negative (reference)				
Close/Uncertain	1.242	0.81	1.903	0.32
Positive	1.793	1.038	3.096	0.036
**Overall survival**				
**Age**	1.059	1.047	1.071	<.001
**Alcohol**				0.007
Never (reference)				
Yes	1.365	1.088	1.711	0.007
**Period of treatment**				0.001
1998–2002 (reference)				
2003–2007	0.731	0.548	0.975	0.033
2008–2012	0.621	0.46	0.838	0.002
2013–2017	0.589	0.407	0.85	0.005
2018–2022	0.352	0.17	0.731	0.005
**T category**				<.001
T1 (reference)				
T2	2.161	1.634	2.856	<.001
T3	2.607	1.93	3.521	<.001
T4	3.074	1.792	5.273	<.001
**N category**				<.001
N0/Nx (reference)				
N1	1.389	0.862	2.24	0.178
N2	2.152	1.513	3.06	<.001

## Discussion

To the best of our knowledge, this is the first study analyzing in detail the sex differences in a series of patients with laryngeal cancer treated with CO2-TOLMS at a single academic center with a long-term follow-up. As expected, the percentage of women treated increased over time in the past 20 years, rising from 6 to 13%. This finding, also accompanied by an increase in the incidence of cases, confirms previous epidemiological studies that advised of the progressively increasing risk of laryngeal cancer in the female population [5]. Of interest, and contrary to our initial hypothesis, no differences could be observed regarding oncologic and functional outcomes between sex groups in our sample. However women were diagnosed at an earler stage and had fewer complications than men after treatment.

Interest in sex differences and cancer treatment has been growing among researchers. A recent study warned that cancer treatments caused more serious adverse effects in women than in men. The study included more than 23,000 patients who participated in phase II and III clinical trials of cancer therapies over 30 years. The authors reported that chemotherapy, targeted therapies, and immunotherapy posed up to a 34% higher risk of side effects, both symptomatic and objective, compared to men. The risk raised to 49% in immunotherapy treatments [26].

In the field of HNSCC, sex disparity has also received attention [27]. Benchetrit et al. reported that women were underrepresented in HNSCC chemotherapy clinical trials cited by the national guidelines. Specifically, according to a recent review, only 5% of HNSCC studies mention sex or gender as a planned analytical variable [28]. Additionally, women were less likely than men to receive definitive chemoradiotherapy as opposed to definitive radiotherapy [28].

In 2019, Park et al. compared cancer mortality and treatment outcomes between women and men. They used a generalized competing event model that controlled for age, sex, tumor site, surgical treatment, and Charlson Comorbidity Index (CCI), to determine the relative hazard for cancer mortality [29]. Women were less likely to receive intensive treatment than men, the cumulative incidence of cancer-specific death was higher for women than men, and women had a lower rate of competing mortality in comparison to men. The authors hypothesized that unmeasured factors, including an implicit physician bias and variation in patient treatment goals, could have contributed to the lower utilization of intensive therapy. Similar sex disparities have been reported for other locations and types of cancer with alike outcomes [30–32]. In our study, no treatment bias was established for treatment indication between genders, and cancer-specific death was similar in both groups after correcting for bias. Moreover, this is the first study that has specifically evaluated transoral surgical treatment with laser in laryngeal cancer; thus, our results cannot be compared with previous literature.

Li et al. analyzed data extracted from the Surveillance, Epidemiology, and End Results Database for patients with laryngeal squamous cell carcinoma (2004–2013) to evaluate modality treatment and outcomes according to sex [33]. The authors concluded that women with T4 disease were more likely to undergo primary radiation (56.8% vs. 45.3%; *p* < 0.001) and less likely to undergo open surgery than men (37.1% vs. 48.2%; *p* < 0.001). In their study, women had significantly better OS in both, glottic and supraglottic cancers, and comparable survival in the subglottic location. Other authors have also reported superior survivals in females with HNSCC than in males (34–35). However, in a recent meta-analysis, Locatello et al. [12] observed no significant difference in survival outcomes, regardless of the survival endpoint (including OS and DSS).

In our study, a slight reduced 5y-OS was found in men compared to women, although it did not reach statistical significance. In any case, these results should be evaluated with caution, because the total number of women in the study was small, and the perhaps with a higher number, differences could have been significant.

A recent analysis based on the United States of America National Cancer Database (NCDB) found that women are less likely to have positive margins after TOLMS [36]. Conversely, in our study, neither margins nor laryngeal exposure did vary significantly according to sex group. Moreover, period of treatment, that can be seen as a surrogate of team experience, was and independent factor for survival, and responsible of some discordant results in the univariate analysis, since the number of women increased in the last years.

Some authors have justified a higher survival rate in women due to the existence of a better social support network, to the evidence that women are more likely to seek medical care, and to the fact that females have, in general, better adjustment than males after treatment (37–38). This seems to be the case in our sample, as women tended to seek attention at an earlier phase of the disease and cancers were then treated at an earlier stage than in men.

A recent study evaluated the summatory effect of gender and race on overall survival in HNSCC. Females with non-oropharyngeal HNSCC had better five-year overall survival than males (56.3% versus 54.4%, respectively), though Black females (52.8%) had poorer survival than both White (56.2%) and Hispanic (57.9%) males [39]. Similarly, Molina et al. sought to determine the impact of race, ethnicity, and socioeconomic status on patients with oral cavity and larynx cancers [40]. Independent predictors of poorer outcomes were race, poverty, age, sex, tumor site, stage, grade, treatment modality, and a history of smoking and alcohol consumption. The authors advised that earlier diagnosis and greater access to surgery and adjuvant therapies in the group of African Americans and poorer would yield significant improvement in outcomes.

Ethnic differences were not a critical issue in our center, most patients were white men and women, and our public health system has universal coverage. However, earlier diagnosis in women turned out to be responsible for higher survival rates and larynx preservation in our sample. These findings agree with Molina et al. [40] who reported that education, poverty, and social support were relevant prognostic factors of survival.

Because of differences in larynx anatomy, we expected a more difficult approach to the larynx in females compared to males. However, no significant differences were found neither in exposure rates during surgery nor in the percentage of negative margins. Moreover, the number of complications was reduced in the group of women which, in turn, yielded in a shorter hospital stay. Also, the need for adjuvant treatment or the presence of local relapse during the follow-up was lower in females, which may suggest a good accomplishment of the initial treatment plan.

Contrary to what was expected, we could not demonstrate a different functional outcome according to sex group, being the postoperative voice and deglutition process, and the rate of tracheostomies or gastrostomies similar between gender.

Apart from surgery, little is known about sex differences in other treatments used to treat larynx cancer, for example in radiotherapy response or in its complications. Yang et al. examined the gender influence on the incidence of carotid stenosis (CS) and the impact of microinflammatory findings in laryngeal cancer patients treated with radiation therapy [41]. In their study, women undergoing radiotherapy were less likely to have CS than men. In our series, the number of patients requiring adjuvant treatment was very low and mostly limited to some supraglottic tumors and subsequently no conclusions can be derived from them.

Among the limitations of the study are its retrospective analysis and the reduced number of women. It represents the difference in the incidence of this type of tumors rather than differences in treatment plan or follow-up. We overcome this difficulty performing a propensity score analysis, that helped us to avoid a relevant number of confounding factors. Moreover, the present study was possible in part due to the large number of patients treated in our center, in a country with one of the highest incidences of larynx cancer in Europe.

The strengths of the study are the large number of consecutive patients treated at a single institution, with the advantage that the variables were prospectively and weekly updated in our database. Moreover, no differences were established in the tumor board regarding indications of treatment, and all patients were submitted to the same surgical principles and follow-up.

## Conclusions

CO_2_-TOLMS is a valid treatment for larynx cancer in women with outcomes equivalent to those achieved in the male population. Female sex was not an independent prognostic factor of survival after CO_2_-TOLMS in our sample. More studies are needed to confirm these preliminary results.
